# Adjuvant-Mediated Epitope Specificity and Enhanced Neutralizing Activity of Antibodies Targeting Dengue Virus Envelope Protein

**DOI:** 10.3389/fimmu.2017.01175

**Published:** 2017-09-25

**Authors:** Denicar Lina Nascimento Fabris Maeda, Milene Tavares Batista, Lennon Ramos Pereira, Mariana de Jesus Cintra, Jaime Henrique Amorim, Camila Mathias-Santos, Sara Araújo Pereira, Silvia Beatriz Boscardin, Sandriana dos Ramos Silva, Eliana L. Faquim-Mauro, Vanessa Barbosa Silveira, Danielle Bruna Leal Oliveira, Stephen Albert Johnston, Luís Carlos de Souza Ferreira, Juliana Falcão Rodrigues

**Affiliations:** ^1^Vaccine Development Laboratory, Department of Microbiology, Institute of Biomedical Sciences, University of São Paulo, São Paulo, Brazil; ^2^Center for Innovation in Medicine, Biodesign Institute, Arizona State University, Tempe, AZ, United States; ^3^Center of Biological and Health Sciences, Federal University of Western Bahia, Bahia, Brazil; ^4^Department of Parasitology, Institute of Biomedical Sciences, University of São Paulo, São Paulo, Brazil; ^5^Pasteur Institute, São Paulo, Brazil; ^6^Laboratory of Immunopathology, Butantan Institute, São Paulo, Brazil; ^7^Clinical and Molecular Virology Laboratory, Department of Microbiology, Institute of Biomedical Sciences, University of São Paulo, São Paulo, Brazil

**Keywords:** heat-labile toxins, labile toxins, adjuvants, dengue virus, envelope protein, vaccines, antibodies, immunosignature

## Abstract

The heat-labile toxins (LT) produced by enterotoxigenic *Escherichia coli* display adjuvant effects to coadministered antigens, leading to enhanced production of serum antibodies. Despite extensive knowledge of the adjuvant properties of LT derivatives, including *in vitro*-generated non-toxic mutant forms, little is known about the capacity of these adjuvants to modulate the epitope specificity of antibodies directed against antigens. This study characterizes the role of LT and its non-toxic B subunit (LTB) in the modulation of antibody responses to a coadministered antigen, the dengue virus (DENV) envelope glycoprotein domain III (EDIII), which binds to surface receptors and mediates virus entry into host cells. In contrast to non-adjuvanted or alum-adjuvanted formulations, antibodies induced in mice immunized with LT or LTB showed enhanced virus-neutralization effects that were not ascribed to a subclass shift or antigen affinity. Nonetheless, immunosignature analyses revealed that purified LT-adjuvanted EDIII-specific antibodies display distinct epitope-binding patterns with regard to antibodies raised in mice immunized with EDIII or the alum-adjuvanted vaccine. Notably, the analyses led to the identification of a specific EDIII epitope located in the EF to FG loop, which is involved in the entry of DENV into eukaryotic cells. The present results demonstrate that LT and LTB modulate the epitope specificity of antibodies generated after immunization with coadministered antigens that, in the case of EDIII, was associated with the induction of neutralizing antibody responses. These results open perspectives for the more rational development of vaccines with enhanced protective effects against DENV infections.

## Introduction

Adjuvants are essential components of vaccines, particularly those composed of purified antigens, which naturally have lower immunogenicity than live attenuated microorganisms. Adjuvants are known for their ability to enhance the magnitude of adaptive immune responses, particularly antibody responses, to coadministered antigens; they may impact the longevity, antigen avidity, and modulation of isotype and IgG subclass switches (1, 2). To date, only a limited number of adjuvants have been incorporated into human vaccines, but several different substances, including bacterial toxins, are known for their strong and rather versatile adjuvant effects in various mammalian hosts (3, 4). We are investigating the changes in epitope recognition elicited by adjuvants.

Heat-labile toxins (LT) naturally produced by some enterotoxigenic *Escherichia coli* strains are enterotoxins endowed with potent adjuvant effects when coadministered with purified soluble or particulate antigens after delivery *via* different parenteral or mucosal routes (5–9). LTs, similarly to cholera toxin, are composed of one toxic A subunit with enzymatic ADP-ribosylation activity and a B subunit pentamer that binds to host cell receptors (10, 11). LT adjuvant effects, as well as those associated with *in vitro*-generated non-toxic LT derivatives, including purified B subunits and mutated LT forms, have primarily been ascribed to the activation of dendritic cells and B and T lymphocytes, leading to enhanced mucosal and serum antigen-specific antibody responses (6, 12–19). In addition, previous evidence indicated that LT derivative may modulate the epitope specificity of antibodies raised against coadministered antigen (12). Indeed, adjuvant-mediated modulation of epitope specificity of antibodies is still a poorly understood issue, particularly in situations involving the design of vaccines capable of inducing protective immunity to various infectious diseases.

Dengue fever is caused by an arbovirus transmitted by *Aedes aegypti* mosquitoes. Fifty to one hundred million people are affected by the disease in tropical and subtropical regions annually, and thousands are potentially at risk to develop more serious side effects, which may lead to death caused by the illness (20, 21). The dengue virus (DENV) has an envelope composed of a single protein (E) and encodes two other structural (prM and C proteins) and seven non-structural proteins (22–24). The glycoprotein E is composed of three structural and functional domains that mediate the initial steps of the DENV infectious cycle. Receptor binding is mainly triggered by domain III [envelope glycoprotein domain III (EDIII)], which leads to the subsequent entry and replication of virus into susceptible host cells (22, 24, 25). EDIII-specific antibodies can confer protection to DENV infection and have been considered protection correlates for anti-DENV vaccines (26–31). Nonetheless, depending on the specificity, concentration and affinity of antibodies targeting the E protein, antibodies may either block virus infection or promote enhancement of infection (the *antibody-dependent enhancement* effect—ADE) *via* cell entry mediated by Fc-γ receptors (32–35).

Proteomics platforms such as microarrays have been exploited as promising high-throughput assays to measure humoral immune responses, assisting our understanding of the subtle characteristics of successful immunization, a key challenge in the rational development of effective vaccines (36–38). The immunosignature technology is a microarray-based platform that uses unnatural randomized-sequence peptides, spotted in high density, as a universal and robust assay to identify antibody-binding patterns of natural or induced responses in both humans and mice. Due to its successful applicability for both infectious and non-infectious diseases, this platform can be used as a powerful tool for systematic studies of various vaccine approaches (39–46).

In this study, we compared the adjuvant effects of alum, LT and LTB in vaccine formulations containing a recombinant form of the DENV EDIII antigen. The results demonstrate that, in contrast to mice immunized with the alum-adjuvanted formulation, mice parenterally immunized with EDIII admixed with LT or LTB mounted a more efficient antibody response capable of inhibiting DENV infection. Immunosignature analysis of the humoral immune response elicited in vaccinated mice showed that LT differentially modulates the epitope specificity of the EDIII-specific IgG antibodies. In addition, the results led to the identification of an EDIII-derived peptide specifically involved in the infectivity of the virus into host cells. Together, this study emphasizes the role of adjuvants in the modulation of the epitope specificity of antibodies induced upon vaccination and, thus, may impact the development of anti-DENV and other subunit vaccines.

## Materials and Methods

### Cloning of Adjuvant LTB

LTB was obtained after cloning the nucleotide sequence of the *eltB* gene (encoding the B subunit of the LT from H10407 strain) into the pET22b vector. The *eltB* sequence (450 bp) was amplified by PCR using the forward primer 5′-CAGAAGGCGGAATGACATATGAATAA-3′ and reverse primer 5′-TTAAAAGCATGTCTCTCGAGAGAACC-3′ (the underlined sequences indicate the *Nde*I and *Xho*I restriction sites, respectively). The insert generated by the PCR reaction was digested and ligated into the corresponding *Nde*I and *Xho*I restriction sites of the pET22b expression vector (Novagen-Merck Bioscience, MA, USA), generating the recombinant plasmid pET22bLTB, which was subsequently used to transform chemically competent *E. coli* DH5α. Transformants were analyzed by digestion with the enzymes *Nde*I and *Xho*I. The recombinant DNA fragment was sequenced and compared to data reported for the LT sequence (GenBank database GI: 408994). A chemically competent *E. coli* BL21 (DE3) strain was transformed with the pET22bLTB plasmid and denominated as *E. coli* BL-LTB.

### Expression and Purification of Adjuvants

The LT and LTB adjuvants were obtained from *E. coli* strains harboring the plasmid pML19 (47, 48) or pET22bLTB, respectively. The purification of LT was based on a previously described method (47). The recombinant *E. coli* strains were cultivated in Luria-Bertani (LB) medium or Terrific Broth (TB) supplemented with 100 µg/ml of ampicillin at 37°C in an orbital shaker (200 rpm). While LTB expression was obtained after 0.75 mM IPTG induction of the *E. coli* BL-LTB strain cultures for 6 h, LT production was achieved by constitutive expression of the *eltAB* gene in overnight cultures of a pML19-harboring *E. coli* strain. The cell pellets were suspended, and the bacterial cells were disrupted by high pressure in a homogenizer (APLAB-10 model, ARTEPEÇAS, Brazil). The LT and LTB contained in the bacterial cell homogenates were purified by affinity chromatography on immobilized d-galactose columns (Pierce, Waltham, MA, USA) previously equilibrated with TEAN buffer (50 mM Tris, 1 mM EDTA, 3 mM NaN_3_, and 0.2 M NaCl, pH 7.4) in an Akta FPLC (Amersham Pharmacia Biotech, Buckinghamshire, UK). The elution step was performed with TEAN supplemented with 0.3 M d-galactose. The proteins were monitored by 15% sodium dodecyl sulfate-polyacrylamide gel electrophoresis (SDS-PAGE). The concentrations of the purified proteins were determined by absorbance measurements in a spectrophotometer (Gene Quant spectrophotometer GE Amersham Biosciences, Buckinghamshire, UK) as previously described (49).

### Cloning, Expression, and Purification of Antigen

The pE1D2 plasmid (50), harboring gene encoding the ectodomain of the envelope glycoprotein from the DENV2 New Guinea C (NGC) strain, was kindly provided by Dr. Ada Alves and used as template for PCR reactions. The sequences of the forward and reverse primers used in PCR reactions were 5′-ACATGCGAGGATCCGGAATGTCATACTCTAT-3′ (the underlined sequence indicates the *Bam*HI restriction site) and 5′-GCCTTCTACTCGAGTTACGATAGAACTTCCTTTCTTA-3′ (the underlined sequence indicates the *Xho*I restriction site), respectively. The PCR product was introduced into pET28a(+) expression vector (Novagen, Germany), generating the recombinant plasmid pDEDIII. The *E. coli* BL21 (DE3) strain harboring the plasmid pDEDIII was cultivated in LB medium containing 50 µg/ml kanamycin. The culture at an OD_600nm_ of 0.5 was induced by IPTG to a final concentration of 0.5 mM and incubated for 4 h. The bacterial pellet was suspended in buffer A (0.1 M Na_2_PO_4_, 0.5 M NaCl, pH 6.8) and lysed in the APLAB-10 homogenizer (ARTEPEÇAS, Brazil). The inclusion bodies were solubilized in buffer A supplemented with 8 M urea, and the recombinant protein was refolded in buffer A. The soluble protein was submitted to nickel affinity chromatography using a HisTrap™ HP column (GE Healthcare Life Sciences, Buckinghamshire, UK). The purified EDIII was evaluated by 15% SDS-PAGE and measured by spectrophotometry.

### Endotoxin Removal from Purified Protein Preparations

The endotoxin was removed using a Detoxi-gel Endotoxin Removing Gel (Pierce, Waltham, MA, USA), and any residual endotoxin was detected by the Limulus Amebocyte Lysate (LAL) QCL 1000 kit (Lonza, Basel, Switzerland). Following treatment for LPS removal, the residual endotoxin contents in the LT, LTB, and EDIII samples were less than 0.1 EU/μg of protein, corresponding to the levels allowed for preclinical research (51).

### Biological Characterization of LT and LTB Adjuvants

The cytotonic activity of the LT derivatives on the adrenal Y1 cells was evaluated as previously described (6). The Y1 cells were seeded in 96-well plates (5 × 10^4^ cells/well) and exposed to 1 µg of LT or LTB, or to phosphate-buffered saline (PBS) as negative control, diluted in Dulbecco’s modified Eagle’s medium (DMEM, Vitrocell Embriolife, Brazil) supplemented with 2% fetal bovine serum (FBS, Vitrocell Embriolife, Brazil). After 8 h of incubation, the cells were observed for the cytotonic effect. The GM1-ELISA was performed as previously described (52) to evaluate LT binding to the cognate ganglioside GM1. Microtiter plates (Polysorp, Thermo Fisher-Nunc, Roskilde, Denmark) were coated with GM1 ganglioside (1 µg/ml) (Sigma-Aldrich, St. Louis, MO, USA) diluted in PBS (pH 7.4) and incubated overnight at room temperature in a humid chamber. Plates were blocked with PBS containing 0.1% BSA. Then, serially twofold diluted LT samples were applied in duplicate wells. To detect LT derivatives, the plates were sequentially incubated with anti-LT serum (titer equal to 10^5^) and horseradish peroxidase (HRP)-conjugated anti-mouse IgG antibodies (Sigma-Aldrich) diluted to 1:1,000 and 1:3,000, respectively, in PBS containing 0.05% Tween-20 and 0.1% BSA. The chromogenic reactions developed with ortho-phenylenediamine dihydrochloride (Sigma-Aldrich) and H_2_O_2_ were stopped with 1 M H_2_SO_4_ and measured at *A*_492nm_.

### Analysis of the Biological Activity of Recombinant EDIII in Vero Cells

This assay was performed as previously described (53). African Green Monkey Kidney Epithelial Cells (Vero Line), which express receptors for E glycoprotein, were cultured in minimum essential medium (MEM) supplemented with 2% FBS and incubated at 37°C in an atmosphere containing 5% CO_2_ for 24 h. Cells were incubated with 100 µg/ml of EDIII protein in MEM at 37°C in 5% CO_2_ for 30 min. After washing with PBS with 2% FBS, Vero cells were fixed with 4% paraformaldehyde (Sigma-Aldrich, St. Louis, MO, USA) for 10 min, treated with anti-EDIII sera harvested from mice immunized with EDIII plus Freund’s adjuvant for 60 min and subsequently with goat anti-mouse IgG conjugated to FITC (Invitrogen, Paisley, UK) for further 60 min. The FITC-stained Vero cells were detected by BD LSRFortessa flow cytometry (BD Bioscience, San Jose, CA, USA), and the data were analyzed using the FlowJo v10 program.

### Animal Immunizations

All experiments involving mice were approved by the Committee on the Ethical Use of Laboratory Animals of the Institute of Biomedical Sciences (CEUA 198) at the University of São Paulo (USP), in accordance with the guidelines for the care and use of laboratory animals adopted by the National Council of Animal Experimentation (CONCEA). The mice were obtained from the Isogenic Mouse Breeding Facility of Department of Parasitology, Institute of Biomedical Sciences—USP. Female BALB/c mice (6–8 weeks old) were immunized *via* the subcutaneous (s.c.) route at days 0, 14, and 28 of the immunization protocol with the vaccine formulations. Groups of five BALB/c mice were treated with 100 µl of PBS alone as a negative control or PBS containing 10 µg of EDIII with or without one of the following adjuvants: 1.0 µg of LT, 3.2 µg of LTB, or 12.5 µg of Al(OH)_3_ (Rehydragel, Reheis, NJ, USA). Serum samples were harvested 2 weeks after each vaccine dose and stored at −20°C until immunological analyses were conducted.

### ELISA

ELISA for anti-EDIII antibodies was performed as previously described (53). Microtiter plates (Maxisorp Thermo Fisher-Nunc, Roskilde, Denmark) were coated with EDIII (1 µg/ml) diluted in PBS pH 7.4 and incubated overnight at 4°C. For ELISA using denaturing conditions, the EDIII protein was submitted to heat treatment at 100°C for 15 min and used to coat the plates. Plates were blocked with PBS containing 3% gelatin for 2 h at 37°C. The serum samples harvested from mice immunized with EDIII alone or coadministered with each adjuvant were applied to duplicate wells and serially twofold diluted. The serum IgGs were measured with HRP-conjugated anti-mouse IgG (1:3,000), IgG1 (1:10,000), or IgG2a (1:3,000) antibodies (Sigma-Aldrich, St. Louis, MO, USA). Absorbance at 492 nm was obtained as previously described and used to calculate the antibody titers, defined as the highest sample dilution able to generate an *A*_492nm_ of 0.2 above the preimmune sera.

### Purification of Serum IgG Fractions

Anti-EDIII IgG antibody fractions were obtained from serum samples harvested from mice submitted to EDIII-based immunization regimens. First, the recombinant 6×-His-tagged EDIII was immobilized in a column filled with a nickel-coupled resin. After serum delipidation, as previously described (54), the sera were submitted to affinity chromatography on EDIII-immobilized resin using 0.05 M PBS, pH 7.4. The anti-EDIII antibodies were eluted in 0.1 M glycine buffer (pH 4), neutralized with 1 M Tris–HCl (pH 9.0), and dialyzed in PBS (pH 7.4). Subsequently, the anti-EDIII antibodies were subjected to affinity chromatography to protein G sepharose according to the manufacturer’s instructions (GE Healthcare, Buckinghamshire, UK), and the IgG fraction was obtained following the elution step described above. The purified EDIII-specific IgG antibodies were measured by BCA assay (Pierce, Waltham, MA, USA) and monitored by 12.5% SDS-PAGE.

### Affinity Assay for Anti-EDIII IgG Antibodies

The antibody affinity index was measured using a modified ELISA protocol with ammonium thiocyanate (47). Briefly, the plates were coated with EDIII as described above. The sera collected from mice from different immunization groups were tested at dilutions corresponding to an OD_492nm_ of 0.8, while purified anti-EDIII IgG antibodies were evaluated at an OD_492nm_ of 0.5. After incubation with sera or purified antibodies, different concentrations of sodium thiocyanate were added to wells and incubated for 15 min. The plates were washed and incubated with HRP-conjugated anti-mouse IgG antibody. The percentage of antibodies bound to EDIII was determined: OD_492nm_ in the presence of ammonium thiocyanate × 100/OD_492nm_ in the absence of ammonium thiocyanate. The values obtained with the serum samples represent the antibody avidity, which under our conditions depends on the affinity and valence of the immunoglobulins since the parameter of structural arrangement was standardized by the use of the antigen immobilized on the solid support. The analyses of the purified IgG antibodies showing constant Ig valence generated average affinity values for each immunization group.

### Plaque Reduction Neutralization Test (PRNT)

To perform the PRNT, the DENV2 NGC strain, kindly provided by Laura Helen Vera Gonzales Gil (Fiocruz, Recife, Brazil), was purified, and the Vero cells were cultivated as previously described (35). Briefly, the NGC strain was propagated in the *Aedes albopictus* cell line C6/36 cultured in Leibovitz medium containing 5% FBS and grown at 28°C for 7 days. The supernatant of C6/36 infected with the NGC strain was collected and titrated for the determination of PFU/ml. For the neutralization test, Vero cells were maintained in DMEM supplemented with 10% FBS and seeded in 24-well plates (1 × 10^5^ cells/well) 24 h before infection. Serum samples from immunized mice were inactivated for complement proteins for 30 min at 56°C and serially twofold diluted. Purified EDIII-specific IgGs were tested at different concentrations. C6/36 cell supernatants containing 500 PFU of DENV/ml were incubated with each dilution of anti-EDIII sera or purified IgG antibodies for 1 h at 37°C. Vero cells were washed with serum-free DMEM and infected in duplicate wells with 200 µl of the neutralization mixture for 1 h at 37°C. The viral suspension was removed, and the cells were overlaid with 1 ml of complete DMEM [2.5% FBS and 1% carboxymethylcellulose (Synth, São Paulo, Brazil)]. Following incubation of the plates at 37°C for 7 days, the cells were fixed for 15 min with 4% paraformaldehyde and stained with crystal violet for 10 min. The percentage of plaque reduction compared to the positive control (DENV not exposed to antibodies) was calculated. Neutralizing antibody titers were expressed as the serum dilution, or IgG concentration, yielding 50% plaque number reduction (PRNT_50_).

### Immunosignature Assay

For the peptide microarray assay, slides were manufactured using *in situ* synthesis of 330,000 (CIM330K) sequences of non-natural randomized peptides as previously described (40). Statistical analyses of microarray data were performed using JMP 12 software (Statistical Discovery Software from SAS) by importing data analysis from Excel and image-processed data from GenePix Pro-6.0 (Molecular Devices, Sunnyvale, CA, USA). Raw intensities were normalized to each slide by dividing all values per array by the median of that array (median normalization). Poor quality spots were excluded from analysis by flagging them as “absent” upon visual inspection. Values less than 0.01 were set to 0.01, and values from duplicate arrays were averaged and used in the analysis. Important peptides were determined by Student’s two-tailed *t*-test, with Benjamini and Hochberg multiple test correction applied to a *p* value < 0.05. For principal component analysis, we used significant peptides for each vaccine approach and an analysis using JMP 12 software (39). The prediction of recognized epitopes was based on lists of informative peptides compared to the EDIII sequence from the DENV2 strain NGC (AHG97599.1) using GuiTope (55). The amino acid substitution matrix was provided by the peptide library and protein sequence. An inversion weight of 1 was selected. Library subtracted scores were returned using the mean of 10 random samplings of the peptide library and a minimum score cutoff of 8.0. The results were graphed using GraphPad Prism v5 using a moving average of 15.

### Competition Assay for DENV2 Using Peptides

This assay was adapted from a protocol previously described (56, 57). Vero cells (1 × 10^5^ cells/well, on 96-well plates) were cultured in MEM supplemented with 3% FBS and incubated at 37°C in an atmosphere containing 5% CO_2_ for 18 h. Intact or heat-denatured EDIII protein, as well as peptide 47 (369-AEPPFGDSYIIIGVEPGQLK-388), custom-made by GeneScript (Piscataway, NJ, USA), at different molar concentrations, was incubated with the DENV2 strain NGC (multiplicity of infection—equal to 1.0). The mixtures were added to Vero cells at 37°C in 5% CO_2_ for 1 h. The mixtures were removed, and MEM supplemented with 2% FBS was added to each well. Following 18 h of incubation, the culture plates were washed with PBS and treated with 50 µl/well of trypsin (2.5 mg/ml) plus EDTA at 37°C in 5% CO_2_ for 10 min to remove the cells. Vero cells were resuspended with PBS containing 10% FBS and fixed and permeabilized with Cytofix/Citoperm kit (BD Bioscience, San Jose, CA, USA) for 10 min. The cells were incubated for 1 h with mAb 4G2, which recognizes the fusion loop at the extremity of the domain II of the envelope protein (ATCC-HB112), and subsequently labeled with anti-mouse IgG conjugated with Alexa 488 (BD Biosciences) for 30 min. The stained cells were measured by flow cytometry using a BD LSRFortessa (BD Bioscience, San Jose, CA, USA) instrument, and the data were analyzed with FlowJo v10 software to determine the amount of DENV-positive Vero cells. Viral replication analysis was carried out at 24 h post-infection based on a protocol previously described (58). RNA was extracted from 100 µl of infected Vero cell, using a guanidine isothiocyanate phenol method (Trizol LS, Invitrogen), according to the manufacturer’s instructions. The quantitative real-time polymerase-chain-reaction was carried out with a set of primers and probes with FAM as dye reporter for the probe. Primers/probes used in assay for DENV were previously described (58). The assay was performed using the AgPath-IDTM One-Step RT-PCR reagents (Applied Biosystems). Succinctly, we used 5 µl of the extracted RNA in 1 µl of the mix from primers/probe (10 pM/μl) and 19 µl of the reagent mix from AgPath-IDTM One-Step RT-PCR kit following the manufacturer’s instructions.

### Structural Analyses

The amino acid sequence composing the main predicted epitope was indicated in a previously published structural model of the quaternary DENV2 envelope glycoprotein (PDB accession number 1OKE) using PyMol v1.8.4.0.

### Statistical Analyses

The data are represented as arithmetic means ± SD and analyzed for variance (ANOVA) with a subsequent Bonferroni’s multiple comparison test using GraphPad Prism v5 software. For the data generated by the PRNT and affinity assay, the statistical analyses were calculated from two independent experiments, while all other results were analyzed from three independent experiments.

## Results

### LT and LTB Enhance EDIII-Specific Antibody Responses

**Figure 1:**
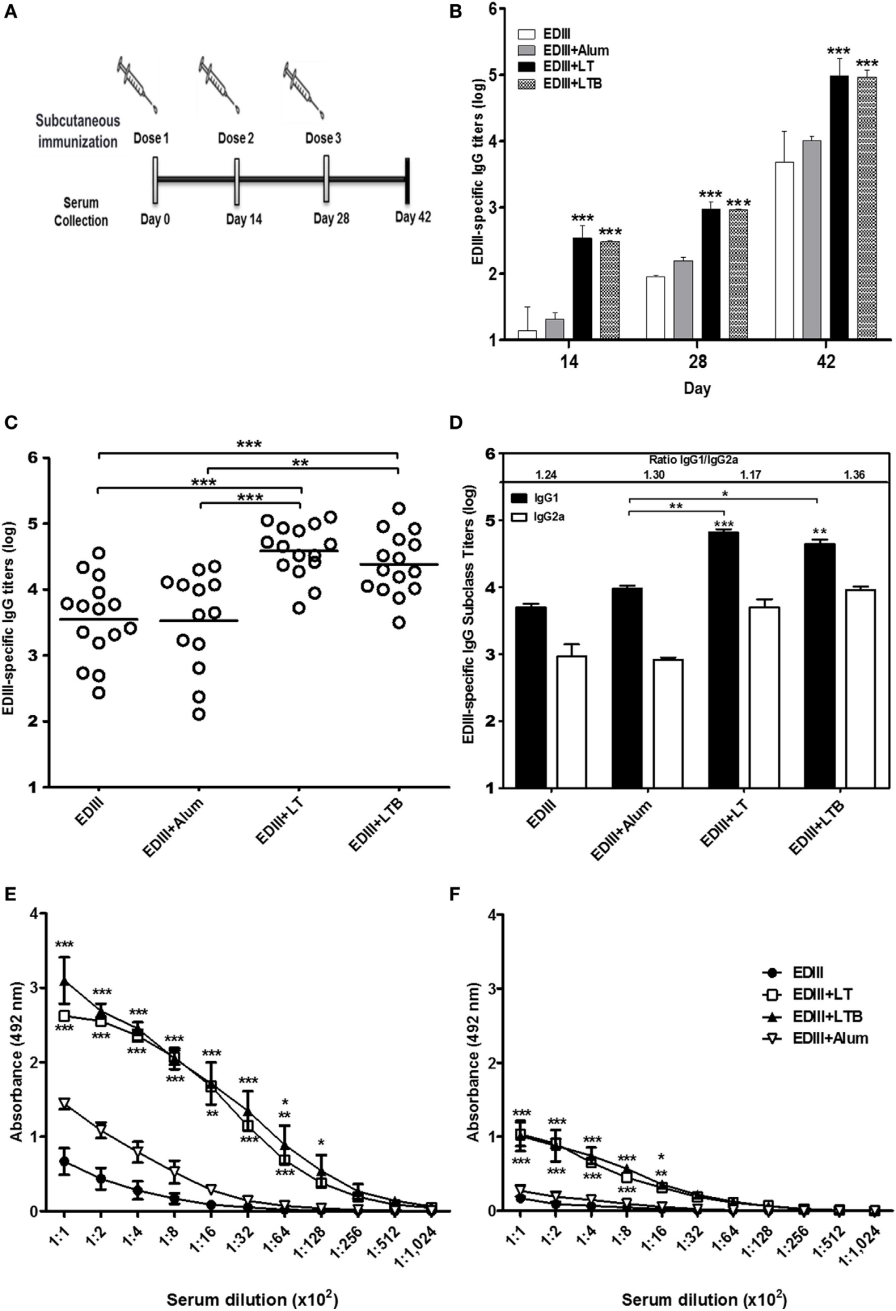
Analysis of envelope glycoprotein domain III (EDIII)-specific antibody responses induced in mice immunized with different vaccine formulations. **(A)** Schematic representation of the vaccine regimen tested in this study. BALB/c female mice were s.c. immunized with three doses of EDIII alone (10 µg) or EDIII coadministered with one of the tested adjuvants [LT1 (1 µg), LT1-B (3.2 µg), or alum (12.5 µg)]. **(B)** Analyses of the antigen-specific IgG responses performed in blood samples collected 2 weeks after each vaccine dose. **(C,D)** Evaluation of the anti-EDIII total IgG **(C)** or IgG subclass **(D)** responses was carried out 2 weeks after the third dose. **(E,F)** Reactivity of serum antibodies carried out by ELISA with the native purified EDIII antigen **(E)** or the same antigen submitted to a heat denaturation treatment **(F)** used as solid phase bound antigens. Anti-EDIII titers were determined in three independent experiments with at least five animals per immunization group. Values represent means ± SD of the IgG titers. ***p < 0.001, **p < 0.01, and *p < 0.05, comparing adjuvanted EDIII-immunized mice to non-adjuvanted EDIII-immunized mice or alum-adjuvanted mice (ANOVA with Bonferroni *post hoc* test).

The recombinant forms of LT, LTB and type 2 DENV EDIII were purified by affinity chromatography (Figures S1 and S2 in Supplementary Material). Both LT and LTB bind to host cell receptors, but as expected, only LT had cytotonic effects to Y1 cells (Figures S1C,D in Supplementary Material). The recombinant EDIII also preserved the receptor-binding function of the native viral protein (Figure S2B in Supplementary Material). Mouse groups were immunized with purified EDIII and vaccine formulations containing EDIII admixed with alum, LT, or LTB. Mice received three s.c. doses at intervals of 2 weeks and were bled one day before each dose and 2 weeks after the last vaccine dose (Figure 1A). Significant increases in the EDIII-specific IgG antibody levels were detected in serum samples of mice immunized with vaccines containing LT and LTB, but not with alum compared to EDIII alone (Figures 1B,C). The antigen-specific IgG1/IgG2a ratios in vaccinated mice were similar, indicating that the adjuvants were not able to modulate the IgG subclass responses induced by the antigen alone. Although anti-EDIII IgG1 and IgG2a titers detected in mice immunized with LT and LTB enhanced in comparison with the non-adjuvanted EDIII, significant differences were only found regarding to IgG1 levels (Figure 1D). In addition, EDIII-specific antibodies in serum samples collected from vaccinated mice were capable of recognizing both conformational and linear epitopes of the antigen, as demonstrated by reactivity with both intact and heat-denatured antigen. Serum samples from mice immunized with LT or LTB showed significantly higher reactivity with both conformational and linear epitopes than those collected from mice immunized with non-adjuvanted EDIII or EDIII admixed with alum (Figures 1E,F).

### Antigen Reactivity and DENV-Neutralization Properties of Purified EDIII-Specific Antibodies

**Figure 2:**
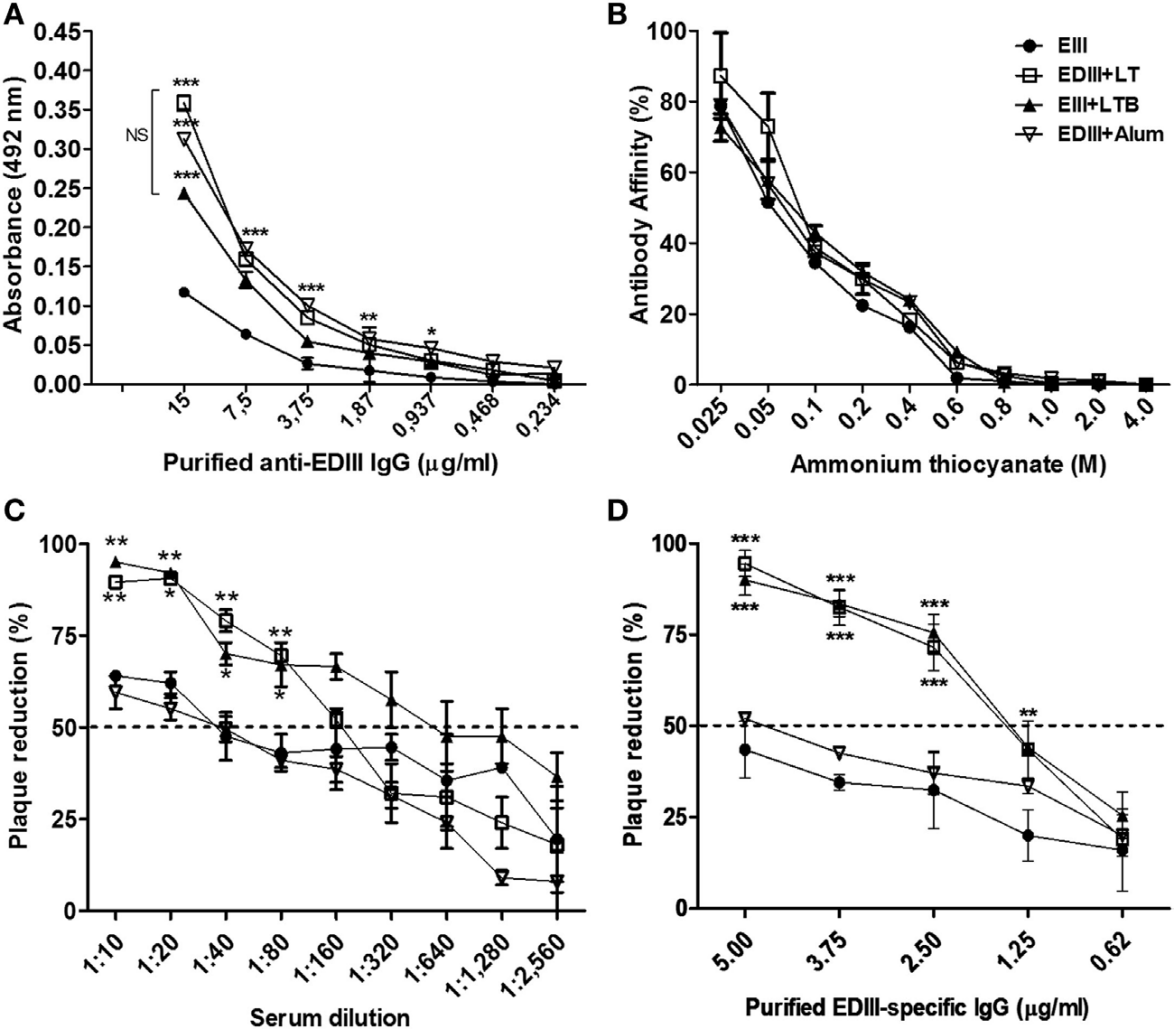
Characterization of purified envelope glycoprotein domain III (EDIII)-specific IgG antibodies raised in mice immunized with different adjuvants. **(A)** Reactivity of antibodies with the purified antigen following ELISA. **(B)** Affinity of purified antibodies to the purified EDIII antigen. Values are expressed by percentage of antibodies that remain bound to the solid phase-adsorbed antigen under presence of ammonium thiocyanate in regard to the reaction performed without ammonium thiocyanate. Immunization groups represented by different symbols as indicated in the insert. **(C,D)** Plaque reduction neutralization test of a DENV2 strain (New Guinea C) carried out with serially diluted serum samples **(C)** or various amounts of purified EDIII-specific IgG antibodies **(D)** derived from mice immunized with the different vaccine formulations, as indicated in panel **(B)**. Results are based on two independently performed experiments carried out with duplicate samples. Values represent means ± SD. ***p < 0.001, **p < 0.01, and *p < 0.05, significant differences with regard to mice immunized with non-adjuvanted EDIII (two-way ANOVA with Bonferroni *post hoc* test). NS: values without statistically significant differences.

To evaluate the role of adjuvants in the modulation of the effector activities of antigen-directed antibodies induced by subunit vaccines, EDIII-specific antibodies raised in different mouse groups were purified by affinity chromatography (Figure S3 in Supplementary Material). The purified anti-EDIII antibodies reacted with the native antigen but did not recognize the heat-denatured antigen, suggesting that the purified antibodies are specifically directed toward conformational epitopes (Figure 2A; data not shown). No difference in antigen affinity was detected in EDIII-specific antibodies raised in the different immunization groups (Figure 2B). Anti-EDIII antibodies, either in serum or purified fractions from vaccinated mice showed virus-neutralization activity *in vitro*; however, anti-EDIII antibodies elicited with LT or LTB were more efficient than those raised with antigen alone or admixed to alum (Figures 2C,D; Table S1 in Supplementary Material). The mice immunized with LTB coadministered to EDIII elicited antigen-specific sera with twofold higher neutralizing capability than LT-adjuvanted vaccination (Figure 2C; Table S1 in Supplementary Material). The purified anti-EDIII IgG from both LT and LTB-adjuvanted mice yielded neutralization titers (PRNT_50_) of 1.6 ng/ml and thereby revealed strong virus-neutralizing effects (Figure 2D).

### Immunosignatures of Anti-EDIII Antibodies

**Figure 3:**
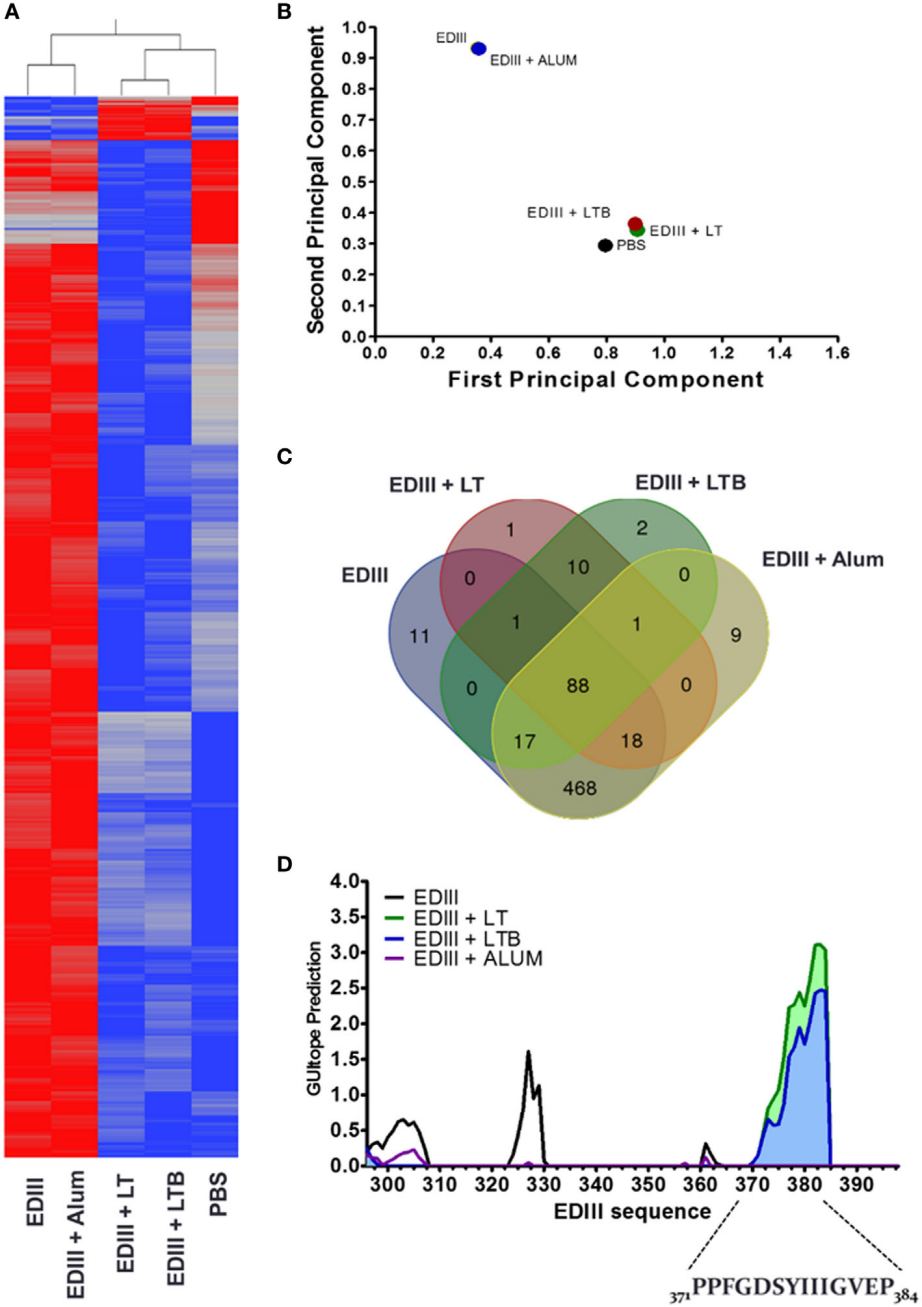
Immunosignature analyses of purified envelope glycoprotein domain III (EDIII)-specific IgG antibodies. **(A)** Heat map demonstrating the immunosignatures detected with EDIII-specific IgG antibodies purified from serum samples collected from mice submitted to the different immunization regimens and a mock mouse group (control). A Student’s *t*-test (*p* < 3.33 × 10^−6^) between vaccine groups was used to select the informative peptides. Hierarchical clustering using EUCLIDEAN distance was used as a measure of similarity to cluster the selected peptides (*x* axis) and vaccine groups (*y* axis). Peptides’ intensity is colored where blue corresponding to low intensity and red to high intensity. **(B)** Variance of immune responses among different vaccine groups. Variation among vaccine groups [pooled sera per group (*n* = 5)] for all significant peptides sequences in the principal component analysis plot, where the first two principal components are plotted and individuals are colored according to the vaccination groups. **(C)** Venn diagram between vaccine groups. Results summarize the overlapping peptides that are significantly different above 1.3-fold change of the mock group versus each vaccine group. **(D)** Antigenic epitope prediction for vaccine groups using random peptide arrays. Significant peptides sequences recognized by antibodies of each vaccine group were submitted to epitope prediction with the GuiTope software and using the EDIII protein sequence from DENV2 New Guinea C (AHG97599.1) as reference. Each line graph represents the GuiTope prediction score for each vaccine group.

To gain a better understanding of the enhanced virus-neutralization activity of the anti-EDIII antibodies raised in mice immunized with different vaccine formulations, we determined the immunosignatures of purified EDIII-specific antibodies raised in different immunization groups (Figure 3). By profiling the antibodies with random linear peptide sequences spotted on microarray slides, we observed that antibodies from mice immunized with EDIII and LT or LTB had distinct immunological signatures for those raised in mice immunized without adjuvant or with alum (Figures 3A,B). In addition, fewer peptides were recognized with high binding intensity by EDIII-specific IgGs adjuvanted with LT or LTB, suggesting an increased specificity of these antibodies (Figures 3A,C). Analysis of peptide-binding profiles indicated that antibodies raised in mice immunized with LT and LTB reacted with 119 peptides sequences each, while antibodies raised in mice immunized only with antigen, or with alum admixed with the antigen, reacted with 605 and 603 peptides, respectively. Among these peptides, only 10 were solely recognized by antibodies raised in mice immunized with LT or LTB and 88 peptides among all groups (Figure 3C).

### Epitopes Predicted by Immunosignatures Are Critical for DENV Infection

**Figure 4:**
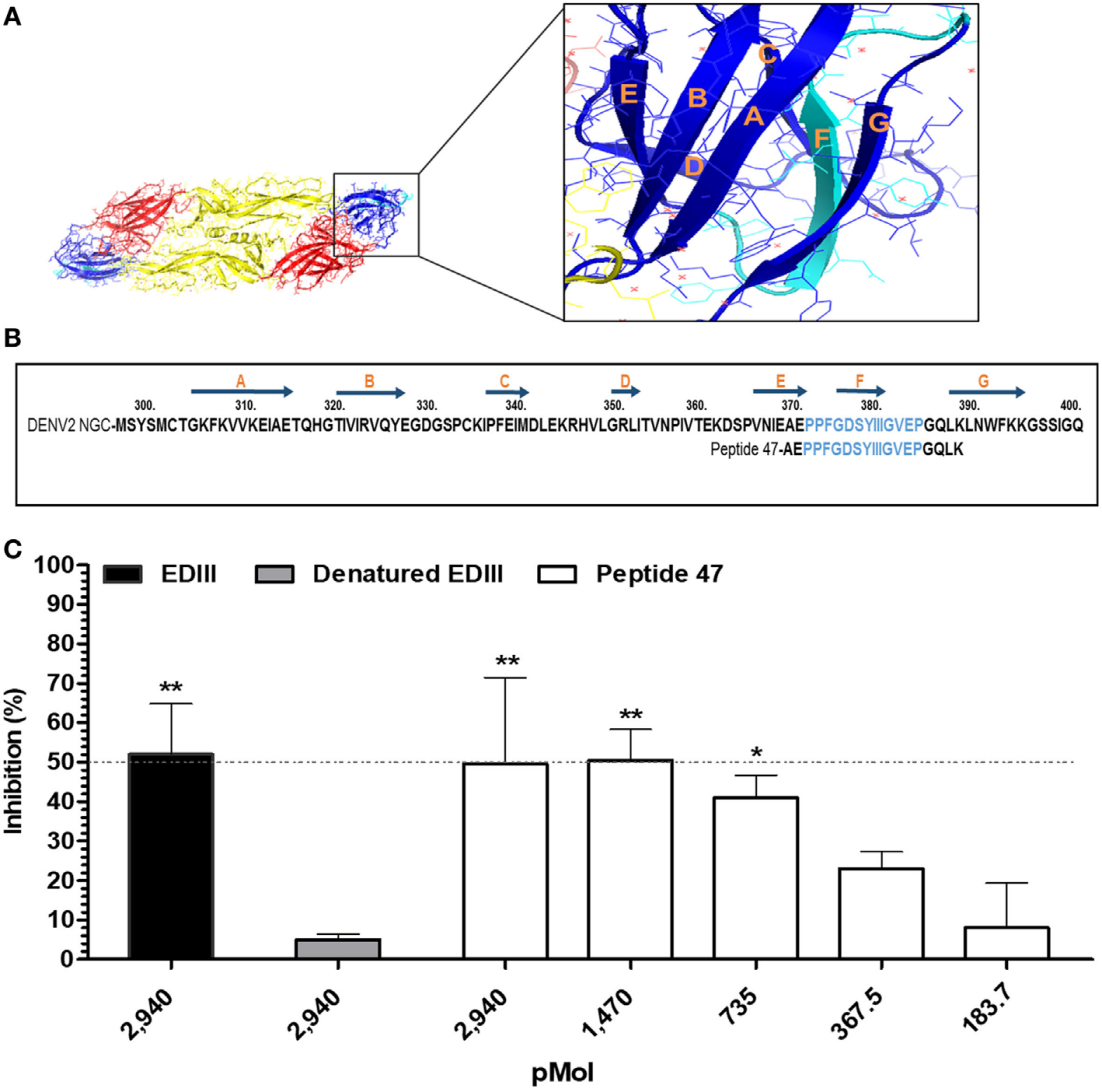
Identification of a linear envelope glycoprotein domain III (EDIII)-derived epitope involved in binding of DENV2 to host cells. **(A)** 3D representation of DENV E protein dimer. Domains I (yellow), II (red), and III (blue) are highlighted. The insert displays EDIII structural features. The amino acid sequence marked in light blue encompasses the epitope recognized by antibodies raised in mice immunized with labile toxins (LT)-adjuvanted vaccines. **(B)** Amino acid sequence of DENV2 EDIII (GenBank accession number: AF038403.1). The positions of the EDIII β strands are indicated above the sequence by dark blue arrows, while the sequences of the synthetic peptide used in the competition assays are shown below. The epitope predicted by the immunosignature is marked in light blue. **(C)** Inhibition of DENV2 infection. Different molar concentrations of synthetic peptide 47 were mixed with DENV2 New Guinea C and subsequently added to cells. Purified EDIII and heat-denatured EDIII were used as positive and negative controls in the inhibition of virus infectivity assay, as indicated in the figure. Cells stained with primary mAb 4G2 and subsequently with Alexa 488-conjugated anti-mouse IgG were measured by cytometry, and the number of DENV-positive infected Vero cells was determined for each group. Values express the reduction in the percentage of the number of virus-infected cells compared to the control group denatured EDIII- (untreated DENV2). Dashed line indicates 50% inhibition of virus infection. Bars represent the mean values and SEM from three independent experiments. ***p < 0.001, **p < 0.001, and *p < 0.05 represent significant differences with regard to denatured EDIII-treated and untreated DENV2 (two-way ANOVA with Bonferroni *post hoc* test).

Prediction of epitopes present in the EDIII antigen and recognized by the different set of purified IgG antibodies showed that mice immunized with both LT derivatives reacted with a specific sequence comprising the EF loop, F beta-sheet, and FG loop between amino acid residues 371–384 (Figures 3D and 4A,B). To further investigate the biological relevance of this finding, we tested a 21-mer synthetic peptide encompassing the complete predicted epitope of the EDIII antigen (Figure 4B). As shown in Figure 4C and Figure S4 in Supplementary Material, the peptide (EDIII_369–388_) efficiently blocked the infectivity of DENV2 NGC strain in Vero cells. A similar inhibition of DENV infectious activity was achieved with the purified EDIII antigen, whereas the heat-denatured antigen lost the virus-infection inhibitory effect.

## Discussion

A considerable amount of information regarding the use of bacterial toxins such as LT as vaccine adjuvants has accumulated during the last four decades. Nonetheless, perspectives on the clinical use of non-toxic LT derivatives have been overshadowed by the induction of undesirable side effects (transient facial paralysis) observed in patients submitted to intranasal immunization trials (59). However, recent observations describing the successful use of LT derivatives as parenterally administered adjuvants rekindled interest in these adjuvants under both experimental and clinical conditions (8, 9, 60–62). In this study, we evaluated the adjuvant properties of LT and a non-toxic derivative composed of the B subunit pentamer as parenterally delivered adjuvants admixed with a recombinant form of the DENV2 EDIII. Our results demonstrated that, unlike alum, LT and LTB enhanced antibody responses to EDIII and induced the generation of antibodies with increased capacity to neutralize DENV2 particles. In addition, we demonstrated that both LT and LTB drastically changed the epitope-binding profiles of EDIII-specific IgG antibodies. Immunosignature analyses performed with peptide microarrays led us to identify an epitope located between the EDIII EF and FG loops, which is specifically recognized by antibodies raised in mice immunized with LT or LTB but not by antibodies raised in mice immunized with alum-adjuvanted EDIII or non-adjuvanted antigen. Remarkably, a synthetic peptide encompassing the epitope identified by the immunosignature analyses interfered with the infectivity of DENV2 in mammalian cells, opening perspectives for therapeutic and preventive interventions. Collectively, this study demonstrated that LT derivatives can modulate the epitope specificity of antibodies after parenteral immunization with purified proteins and thus may contribute to the rational design of subunit vaccines for DENV and other infectious agents.

The evidence presented here demonstrates that LT used as a parenteral adjuvant promotes strong neutralizing antibody responses to DENV infection. Antibody neutralization titers achieved in mice immunized with EDIII and LT or LTB were significantly higher than those elicited in mice immunized with alum. In accordance with these findings, the EDIII-specific IgG fraction purified from mice immunized with LT or LTB showed greater virus-neutralizing activity than antibodies purified from mice immunized with alum. Furthermore, purified IgG collected from mice immunized with LT or LTB revealed a strong virus-neutralizing effect compared to the previously described anti-EDIII mAbs, such as 2H12, 9D12, and 1A1D-2 that block DENV infection under *in vitro* conditions with different intensity (26, 32, 63, 64). The results emphasize the fact that parenterally administered LT represents a promising adjuvant alternative comparable to the most potent adjuvants presently in use under both experimental and clinical conditions. In addition, the observation that purified LT B subunits exert similar adjuvant effects as LT indicated that the local inflammatory reactions induced by the enzymatically active toxin can be abrogated without reducing the immunomodulatory effects to coadministered antigens.

Our results obtained with purified IgGs demonstrated that the specificity, and not the magnitude, of the antibody response is critical in virus neutralization. In fact, antibody-mediated virus-neutralization activity has been strongly correlated with antibody/antigen interaction force and/or epitope recognition patterns (12, 26, 47). In our case, the distinct neutralization effects of the EDIII-specific polyclonal antibodies were not associated with antigen affinity. In accordance with these results, several studies have reported that the virus-neutralizing activity of anti-EDIII mAbs or sera does not necessarily correlate with antigen affinity (33, 35, 64–67). However, epitope recognition patterns of EDIII-specific mAbs showed a positive correlation with virus-infection blockage activity (26, 32–34, 65). In this context, we demonstrated that the immunosignature patterns of antibodies raised in mice immunized with LT derivatives differed drastically from those raised in mice immunized with non-adjuvanted EDIII or alum-adjuvanted antigen. More importantly, such differences correlated with an enhanced virus-neutralizing activity of the IgG antibodies induced by LT forms. Although corroborative evidence remains to be gathered, this study clearly demonstrated that adjuvant-mediated modulation of epitope recognition patterns by the antibodies raised is a critical aspect of vaccine-induced immune responses, particularly with regard to the induction of immunity to DENV.

In this report, the immunosignature analyses of EDIII-specific polyclonal antibodies disclosed that, in contrast to alum, incorporation of LT or LTB to vaccine formulation resulted in antibodies with increased specificity to the epitopes located at the EDIII EF and FG loops in addition to the F β-strand. In support of this concept, our study demonstrated that a synthetic peptide comprising the predicted epitope interfered with the *in vitro* infectivity of DENV, as measured with Vero cells. In addition, the lateral FG loop has been implicated in DENV infectivity both in mosquito and mammalian cells (57, 68, 69). More importantly, part of the EDIII epitope unveiled by the immunosignature analyses, comprising the F β-strand and FG loops, has also been identified as a target epitope for anti-DENV and anti-ZIKV mAbs isolated from high responders in clinical studies with ZIKV-infected individuals (70). Together, these findings emphasize the relevance of the EDIII lateral ridge region as a target for antibodies capable of conferring protection from flaviviruses.

The role of LT derivatives, in the context of active immunization practices, on the modulation of epitope recognition profiles of antibodies has been scarcely investigated. Here, and in previous reports, we have demonstrated that LT derivatives shape the epitope recognition profile of antibodies to different pathogens [(12), unpublished data]. Previous studies have demonstrated that LT derivatives improve the expression of co-stimulatory molecules on B lymphocytes and dendritic cells and, consequently, on prime CD4^+^ T cells, leading to modulation of Th1/Th2/Th17 cytokine production patterns (12, 17, 19, 71–73). Although future studies will have to be performed, the capacity to program B cells using cognate T helper lymphocytes, concomitant with direct activation effects, may represent possible alternatives to explain the action of LT derivatives on the modulation of the epitope recognition pattern of antibodies.

Synthetic peptides have been largely exploited in therapeutic approaches against viral diseases such as hepatitis, acquired immunodeficiency syndrome and influenza (74). However, little effort has investigated the use of peptides for treatment of arboviroses. Anti-dengue peptides under pre-clinical evaluation were designed after *in silico* analyses and target the stem region, hydrophobic pocket or FG loop of the E glycoprotein (57, 69, 75, 76). These peptides showed half-maximal inhibitory concentration (IC_50_) for virus infection or binding to host cells at micromolar levels (57, 75, 76). Here, based on a different experimental approach, we report the identification of a peptide that affects DENV infectivity with an IC_50_ in nanomolar concentration range. In contrast to previous approaches, our results demonstrate that, besides having a direct effect on the host cell/virus interaction, such peptides may be amenable to generated protective antibody responses and therefore find both prophylactic and therapeutic applications.

Epitope mapping based on linear peptides has been a routine technique for the characterization of both monoclonal and specific polyclonal antibodies. The antibody immunosignature approach relies on the use of random peptides microarrays that broader the epitope screening inasmuch not specific to a pathogen or a vaccine, resulting in reduced cost and time. In addition, random peptides can be reactive with antibodies directed against conformational epitopes, which are not possible with conventional peptide microarray (40, 76). In fact, our data showed that even without the specific EDIII sequences on the array it was possible to detect DENV neutralizing peptide demonstrating the applicability of this technique.

In conclusion, our results demonstrated that LT and LTB enhance both the magnitude and the specificity of the antibody responses elicited in mice immunized with an anti-DENV subunit vaccine, promoting changes to the epitope-binding profiles that, in turn, lead to enhanced virus-neutralization effects with regard to alum. Such findings bring relevant insights into the design of vaccine formulations based on purified proteins and the induction of antibodies capable of preventing infections associated with different pathogens.

## Ethics Statement

All experiments involving mice were approved by the Committee on the Ethical Use of Laboratory Animals of the Institute of Biomedical Sciences (CEUA 198) at the University of São Paulo (USP), in accordance with the guidelines for the care and use of laboratory animals adopted by the National Council of Animal Experimentation (CONCEA). The mice were obtained from the Isogenic Mouse Breeding Facility of Department of Parasitology, Institute of Biomedical Sciences—USP.

## Author Contributions

Conceived and designed the experiments: DM, CM-S, SS, LF, and JR. Performed the experiments: DM, MB, LP, MJ, SP, SS, VS, and DO. Analyzed the data: DM, MB, JA, SJ, LF, and JR. Contributed reagents/materials/analysis tools: JA, SB, EM, SJ, LF, and JR. Wrote the paper: DM, MB, LF, and JR. All the authors reviewed the manuscript.

## Conflict of Interest Statement

The authors declare that the research was conducted in the absence of any commercial or financial relationships that could be construed as a potential conflict of interest. The reviewer, KF, and handling editor declared their shared affiliation.
