# Supplementary material for: Adjuvant-Mediated Epitope Specificity and Enhanced Neutralizing Activity of Antibodies Targeting Dengue Virus Envelope Protein
Source: Front Immunol. 2017 Sep 25;8:1175. doi: 10.3389/fimmu.2017.01175 (PMC5622152; doi:10.3389/fimmu.2017.01175)
Supplement: Supplementary file 1 [file Data_Sheet_1.docx]

**
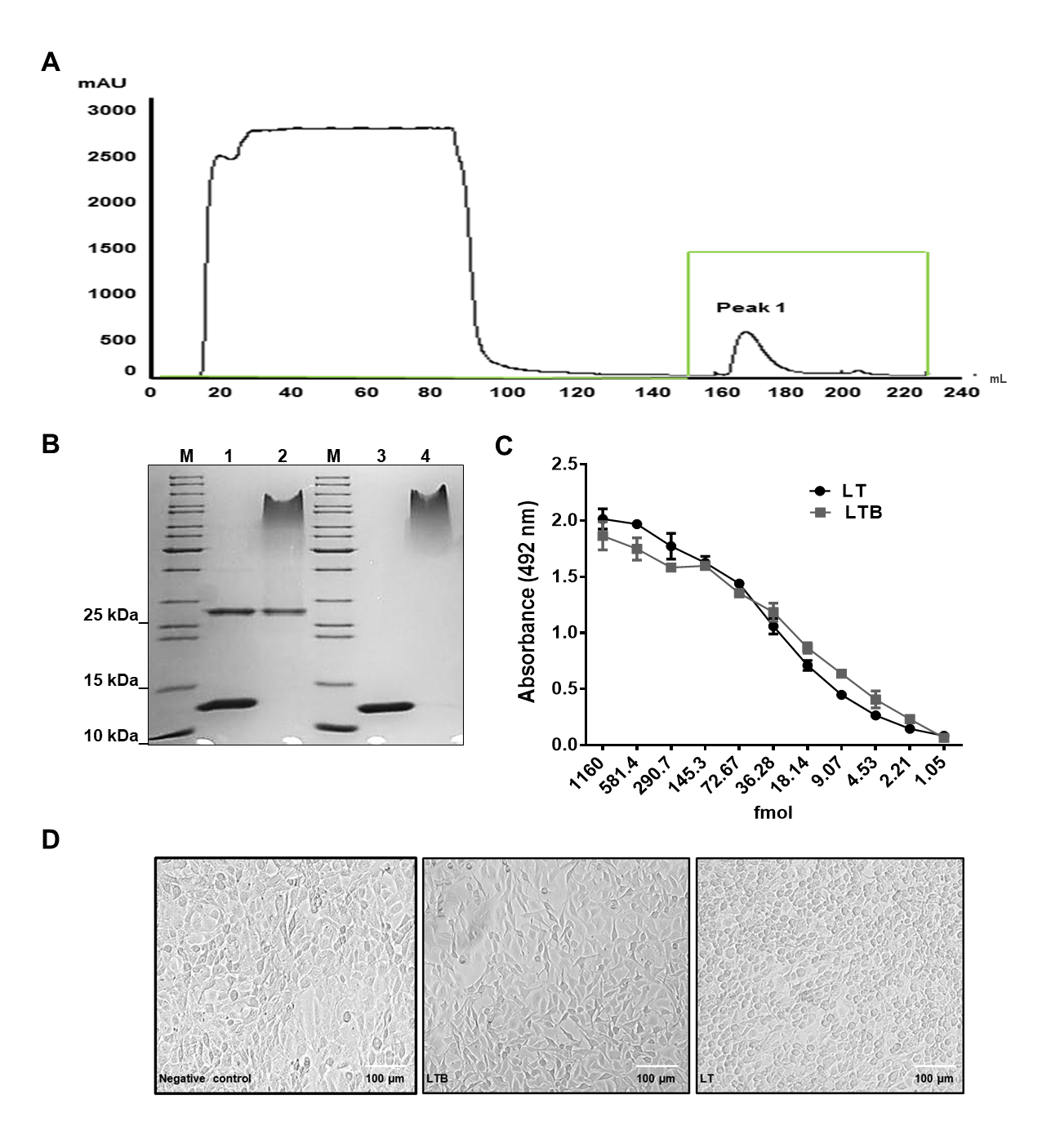
Supplementary Material**

**Figure S1. Purification and biological analysis of LT, LTB and EDIII**. **(A)** Chromatogram of the LTB protein purified by affinity chromatography to D-galactose. The peak 1 corresponds to eluted LTB. **(B)** SDS-PAGE of the proteins purified by affinity chromatography to D-galactose and heated (lanes 1 and 3) or not (lanes 2 and 4) to 100°C prior to loading on the gel. M, PageRuler Unstained Protein Ladder (Thermo Fisher Scientific); lanes 1 and 2, LT (3.0 µg); lanes 3 and 4, LTB (3.0 µg). **(C)** GM1 binding assay of recombinant proteins LT and LTB by ELISA. Equimolar amounts of purified LT (●) and LTB (■) were used. Data are representative of two independent experiments. Values are indicated as means ± SD. **(D)** Y1 cell assay incubated with the LT derivatives showing absence (LTB) and presence (LT) of the cytotonic effect. Negative control represents the cells not treated with LTs.

**
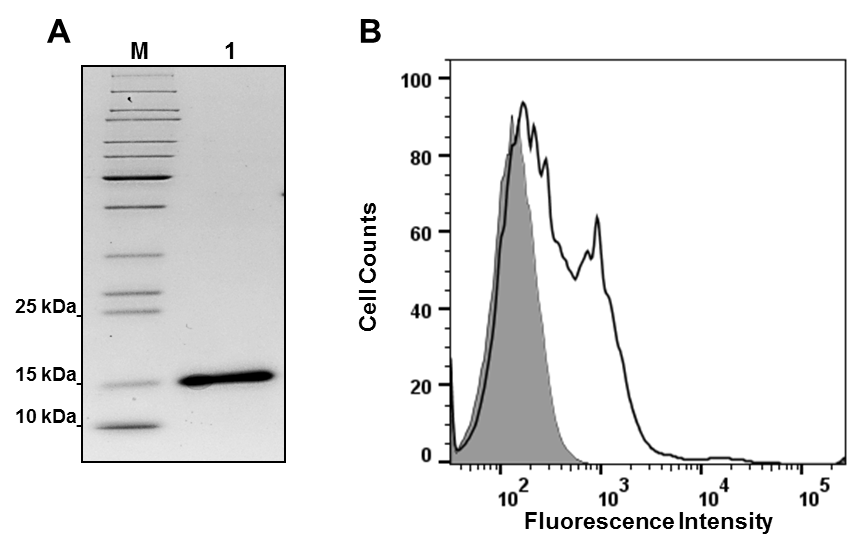
**

**Figure S2. Purification and biological analysis of EDIII. (A)** SDS-PAGE of EDIII protein purified by nickel affinity chromatography. Lane 1, EDIII (3.0 µg) submitted to the heat treatment. **(B)** Binding assay of EDIII to Vero cells measured by flow cytometry. EDIII was marked with mouse anti-EDIII serum and subsequently with anti-mouse IgG antibody conjugated to FITC. Cells incubated with intact (black line histogram) or denatured (filled gray histogram) EDIII.


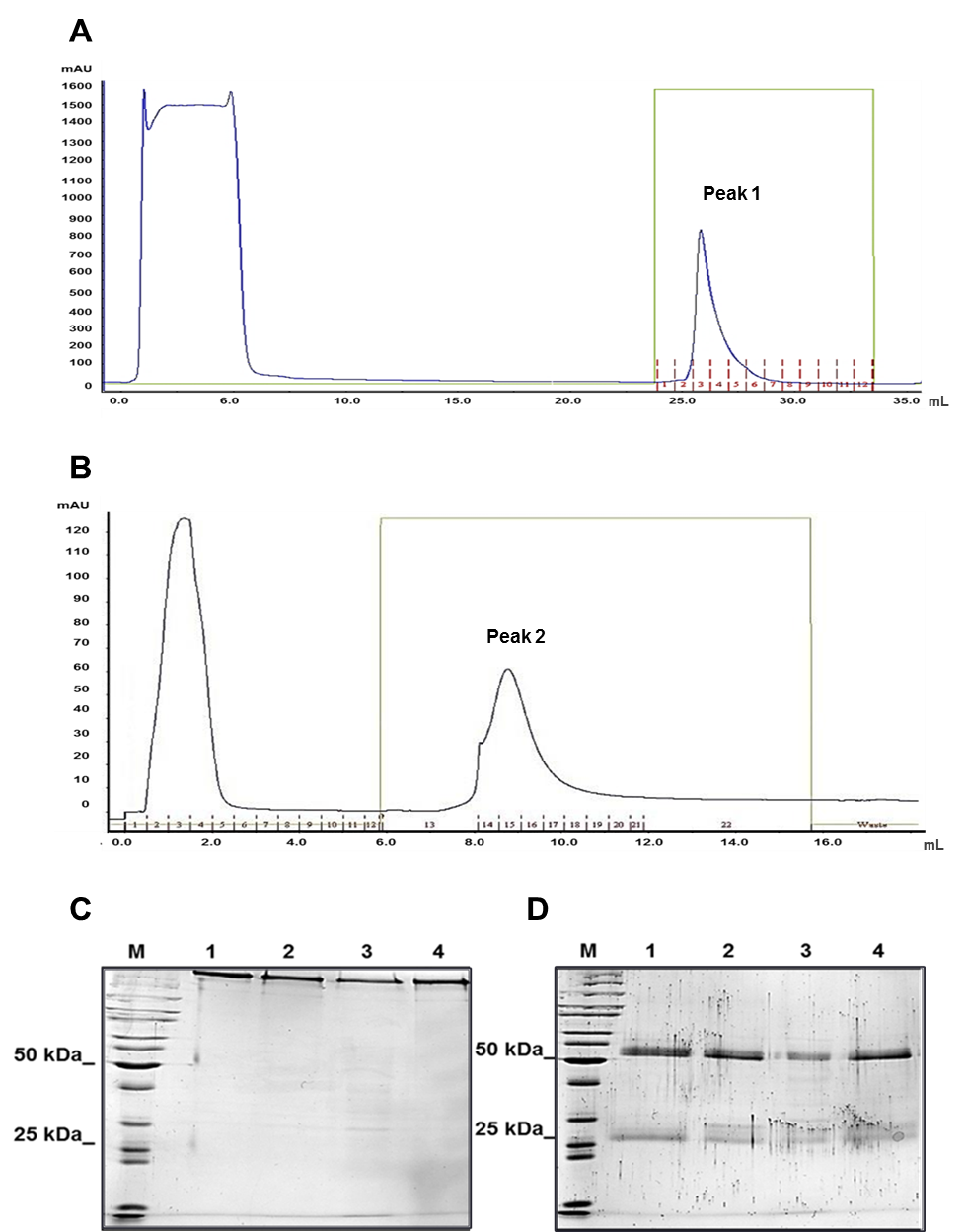


**Figure S3. Purification of the EDIII-specific serum IgG antibodies.** Purified EDIII-specific IgG antibodies were prepared from sera harvested from immunized mice. **(A)** Chromatogram of antigen-specific antibodies purified by affinity to the EDIII immobilized on nickel-coupled resin. **(B)** Chromatogram of EDIII-specific IgG purified by affinity to the protein G. Peaks 1 and 2 correspond to the anti-EDIII antibodies and the anti-EDIII IgG antibodies, respectively, obtained after the elution step with glycine buffer. **(C and D)** Coomassie blue-stained polyacrylamide gel of EDIII-specific IgG antibodies under non-denaturing **(C)** or denaturing **(D)** conditions. EDIII-specific IgG antibodies were purified from sera of different immunization groups: EDIII alone (lane 1) or EDIII in combination with adjuvant LT (lane 2), LTB (lane 3) or alum (lane 4). M, PageRuler Unstained Protein Ladder (Invitrogen).

**Table S1. Neutralizing activity of EDIII-specific serum antibodies.**

**^*^**Represents the highest serum dilution that reduces 50% of the plaque forming units in PRNT.

**^‡^**Values are expressed as PRNT_50_ titer/anti-EDIII serum IgG titer (x10^3^) for each immunization group. Results are means ± SD of two independent experiments.

ᶲ p < 0.01, significant difference with regard to mice immunized with EDIII alone or mixed to alum (ANOVA with Tukey's as post hoc test).

^#^p< 0.05, significant difference with regard to mice immunized with EDIII co-administered to LTB (ANOVA with Tukey's as post hoc test).

|  | **Anti-EDIII IgG Titer** | | **PRNT_50_ Titer*** | **Virus Neutralization Ratio^‡^** |
| --- | --- | --- | --- | --- |
| **EDIII** |  | 6,548.5± 0.087 | 20 | 3.05 ± 0.237 |
| **EDIII+LT** |  | 17,561.7± 0.053 | 160 | 9.11 ± 0.067ᶲ^#^ |
| **EDIII+LTB** |  | 15,468.7± 0.049 | 320 | 20.69 ± 0.09ᶲ |
| **EDIII+Alum** |  | 8,190.2± 0.025 | 20 | 2.44 ± 0.18 |


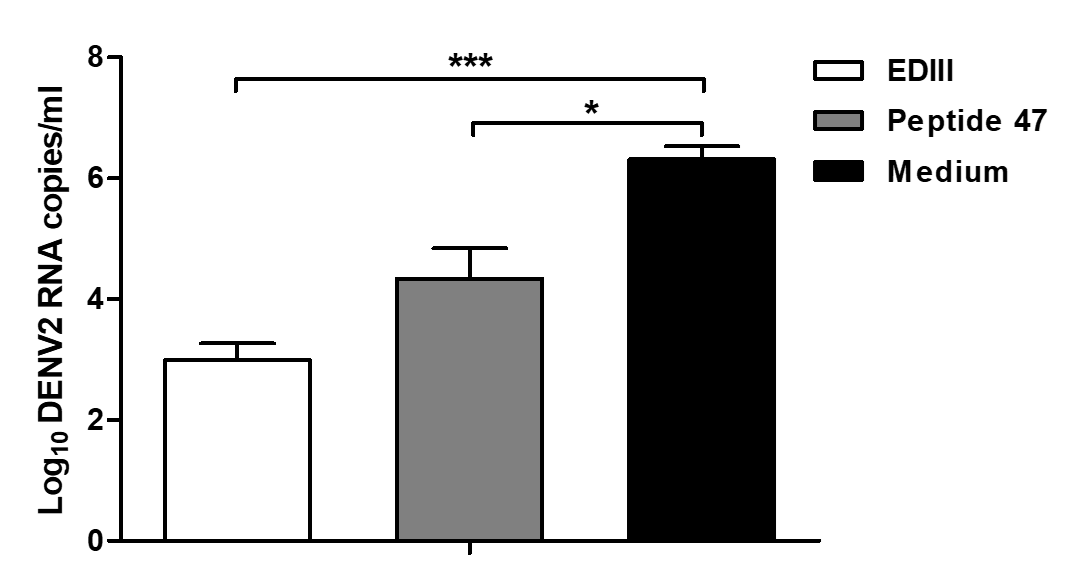


**Figure S4. Inhibition of DENV2 infection**. Vero cells were exposure to DENV2 (MOI = 1) previously incubated or not with EDIII protein or synthetic peptide 47 (2,940 pMol). The DENV2 genome RNA was measured using RT-PCR at 24 h post-infection.
